# First assessment of the biodiversity of praying mantises (Insecta: Mantodea) in Cameroon with DNA barcoding

**DOI:** 10.1371/journal.pone.0304163

**Published:** 2024-05-23

**Authors:** Valeriy Govorov, Evgeny Shcherbakov, Petr Janšta, Barbora Černá Bolfiková

**Affiliations:** 1 Department of Zoology, Faculty of Science, Charles University, Prague, Czech Republic; 2 Faculty of Tropical AgriSciences, Czech University of Life Sciences Prague, Prague, Czech Republic; 3 Faculty of Biology, Lomonosov Moscow State University, Moscow, Russia; Instituto Leonidas e Maria Deane Fiocruz Amazonia, BRAZIL

## Abstract

Praying mantises are the apex insect predators in many ecosystems, nevertheless they receive relatively less recognition in biodiversity reviews. We report a first survey of diversity of praying mantises in Cameroon, which is situated in the Congo Basin region, one of the richest biodiversity hotspots. Combination of light trapping with manual collecting resulted in 495 specimens representing 62 species. A total of eight species are novel for the country, at least five species are likely undescribed. DNA barcodes of 72 specimens representing every collected species were obtained, curated, and submitted to NCBI database. For eight species, barcodes are published for the first time. A maximum likelihood phylogenetic tree was created using all available barcodes of Mantodea of Central African subregion. The results obtained during this study stress the importance of combining traditional and molecular approaches during biodiversity assessments of often neglected taxa, the latter aiding in uncovering new species, resolving unknown morphological divergencies and assigning conspecifics.

## Introduction

Mantodea is an order of predatory insects, currently consisting of about 2500 valid species in 29 families [1]. Praying mantises are widely distributed around the world, however, the highest diversity is accumulated in tropical regions, such as the rainforests of Equatorial Africa. Rich diversity is most likely connected to the Congo Basin forests of Central Africa, the world’s second largest contiguous zone of lowland tropical moist broadleaf forests surpassed only by Amazon basin [2]. The Congo Basin forests account for roughly 20% of the world’s remaining tropical rainforest [3, 4], as well as 70% of the continent’s total plant cover [5, 6] and remain relatively undisturbed. They host a substantial percentage of worldwide biodiversity, as well as represent an irreplaceable part of climate regulation. Despite seeming immenseness, Congo Basin forests are vulnerable to global changes in climate, which are predicted to have a major impact on the region’s biota. Although it has been claimed that Congo Basin forests are particularly resistant to severe drought [7], recent research reveals that a persistent drying trend will reduce its photosynthetic capacity and lead to changes in plant community composition [8], further amplified by the negative consequences of land use changes [9]. Drastic distortion to the environment and to species diversity call for intensification of faunistic research to better plan conservation programs.

Better understanding and deepening existing knowledge of natural mechanisms within this vast but fragile region are closely interconnected with surveying the biodiversity of different groups of organisms. Species diversity quantification is essential especially in the tropics where many species are awaiting description [10, 11]. Particularly, detailed inventory data and sample collection are needed for many taxonomic groups that remain understudied, including invertebrates, to cover existing biodiversity knowledge gaps both within and outside protected areas [12]. Molecular phylogenies not only help clarify the taxonomy of threatened species [13] but also facilitate species listings for monitoring and planning [14], providing a complement to morphological assessment of poorly described taxa [15]. Nowadays, DNA barcodes, standardized short sequences of DNA, are a popular method for quick and accurate recognition of previously known species [16]. Species-specific fragments aid in discovery and delimitation of taxa on any life stage, identification of endangered, invasive and regulated species, as well as help tackle more comprehensive tasks in ecology and conservation biology, such as community assemblies, habitat richness and its suitability for priority protection [17, 18]. Molecular data also aid in pointing out potential weak spots in existing taxonomical consensus and could be used as a prerequisite for further investigations. Such methods are commonly used for studying “dark taxa” [19], small insects that get little taxonomic attention but nevertheless are vital part of ecosystems, including serving as prey items to juvenile mantodeans, thus establishing prey-predator balance. As praying mantises play an important role as consumers, recognizing their species diversity would contribute to further understanding of the ecosystem flow of the infrequently sampled region.

Diversity of Mantodea in Central African region was investigated by just a handful of publications. Roy [20] provided the first comprehensive categorization of praying mantises of Gabon and reported, among other, 25 species considered endemic to central-western forest sector (Cameroon, Republic of the Congo, Equatorial Guinea, Gabon). Later, knowledge of the diversity of praying mantises in Gabon was updated by Moulin [21], based on unpublished collection data and new field collections. Tedrow *et al*. [22] conducted research on Mantodea diversity in several national parks of Rwanda and also pointed out the necessity of taxonomical revision of some groups, requiring more sampling efforts in Rwanda and neighboring regions. Moulin *et al*. [23] published the only currently available barcoding data for Mantodea of Central Africa and established a unique barcode reference database. Their study was conducted based on the collection efforts in Central African Republic (CAR) dating since 1984 and, besides providing a checklist, they pointed out imperfections of systematics of some genera, such as *Cataspilota*, *Plistospilota*, *Miomantis*, *Entella*. A year later, Roy [24] published a checklist of Mantodea of CAR from different site 250 km further northeast and reported greater diversity of genera and species because of more varied biomes.

Among the Central African countries, Cameroon is a high-priority target for biodiversity conservation [25–28]. It has one of the highest species count of mammals (280 species) and vascular plants (9000 species) in Africa, and houses more than 40 globally threatened animal species [29]. Although few diversity studies were conducted in this regard, Cameroon also seems to be a biodiversity hotspot for insects. Despite brief mentions on distribution of about 155 species of praying mantises [30], research dedicated to Mantodea had never been conducted in Cameroon. The southern part of the country is located in the Central African forest, where aggressive deforestation degrades the ecological state of the region [31, 32], making the exploration of the understudied biodiversity of any taxon an increasingly elusive task.

Here we review and assess the Mantodea diversity in studied area of Cameroon. The taxonomic list includes full collection data for all species and comments on selected taxa and is complemented by molecular data. Most importantly, we drastically expand the barcode database of African praying mantises and perform the phylogenetic analysis of the updated dataset.

## Materials and methods

**Fig 1:**
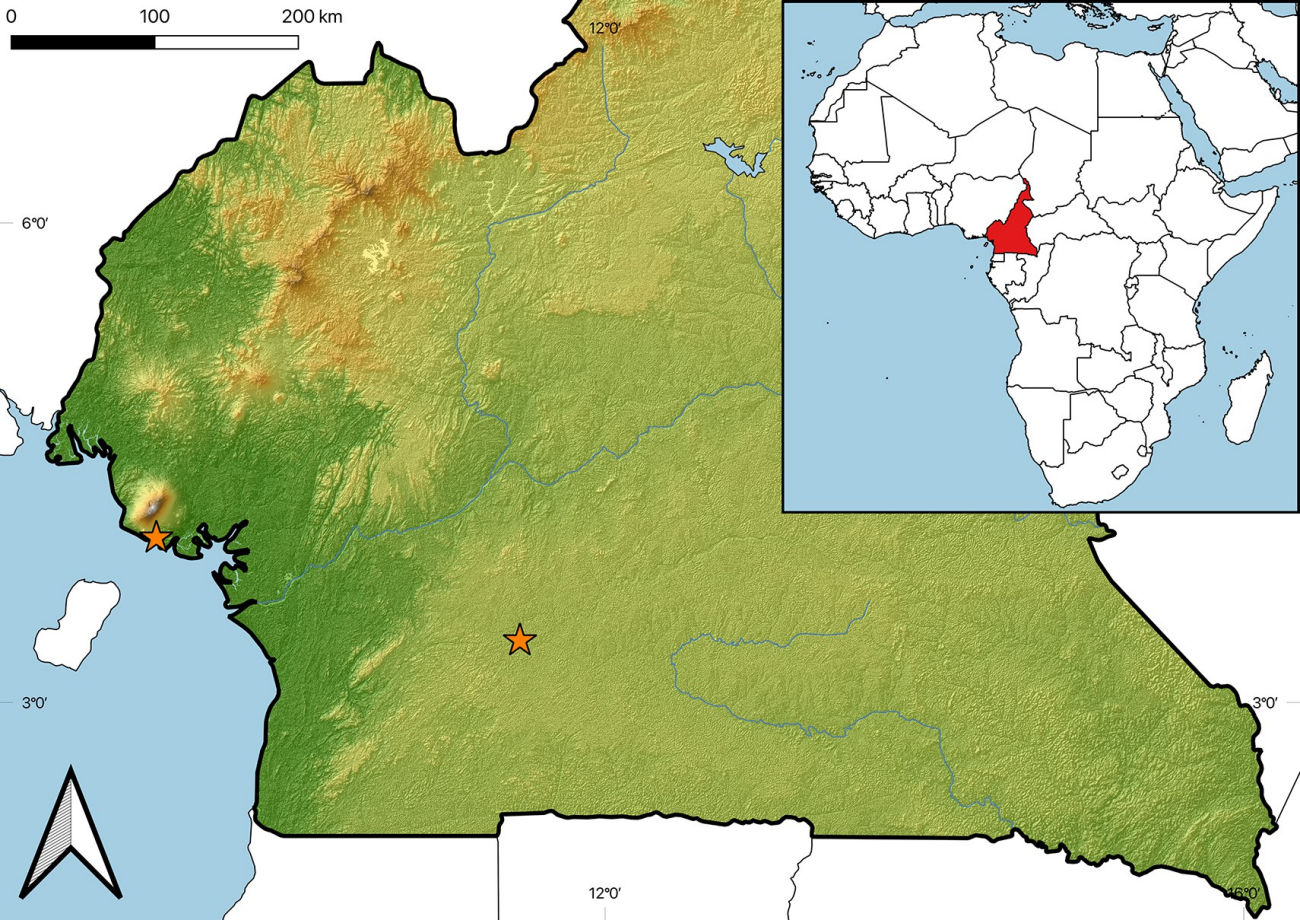
Collecting sites in Cameroon, indicated by orange stars. For detailed coordinates see Taxonomic checklist in Results).

Material collection was conducted in May-August 2021 and January-March 2022, with few individuals collected sporadically in 2015 and 2018 in the village Ebogo II, South Cameroon, 3°23’15.8"N 11°27’59.8"E (see Fig 1). Ebogo II is located in the northern part of the Congo Basin at an elevation around 650 m asl, with a mean annual temperature of 24.3°C. The landscape is relatively flat, with homogeneous natural conditions, originally covered by tropical rain forest. The rainfall, of about 1700 mm on an annual basis, is distributed mostly in two rainy seasons between March and June, and between September and October, respectively. Several specimens of genus *Sphodromantis* were collected in July 2019 near Limbé, South-West Region.

Most specimens were collected using a light trap, consisted of a white sheet illuminated by UV mercury vapor lamp OSRAM HWL (MBFT) 160W during the night. In addition, manual collection efforts were organized in surrounding forest, and included visual examination of vegetation, tree climbing and net sweeping. Insects were fixed using ethyl acetate vapor jar and preserved in 80% ethanol or dried on cotton wraps.

Taxonomy follows the most recent revision of the order [33], while distribution records are given according to Ehrmann [30], Moulin *et al*. [23], Moulin [21] and Roy [24]. Identifications were carried out by first and second authors using dichotomous keys whenever available in comprehensive monographs and dedicated taxon revisions (see Taxonomic checklist in Results). Species that had no recent literature references were identified by studying their original descriptions. When required for species identification, male and female genitalia were separated from the specimens, macerated in 10% KOH solution for 4–36 h and rinsed with water and ethanol afterwards. Prepared genitalia are stored in microvials with glycerol together with corresponding specimens. External morphology and genitalia were studied using Leica M205 C stereomicroscope (Wetzlar, Germany). Photographs of the live specimens were made with Sony Alpha a6000 digital camera (Tokyo, Japan) and Sony SEL30M35 30mm f/3.5 macro lens.

DNA barcoding was performed for a representative number of specimens (n = 72), selected using diversity and material quality criteria. DNA was extracted from tissue (meta- or mesothoracic leg) using DNeasy Blood & Tissue kit (Qiagen) and the isolates were checked for quality and quantity on NanoDrop spectrophotometer. Samples with high DNA concentration (>20–25 ng/μL) were diluted with water until optimal concentration. For 56 samples PCR was done using COH6 (5’- TAD ACT TCD GGR TGD CCA AAR AAY CA -3’) and COL6b (5’- ACA AAT CAT AAA GAT ATY GG -3’) primers [34], the thermal cycle included 1 cycle of initial denaturation at 94°C for 5 min, followed by 30 cycles of denaturation at 94°C for 1 min, annealing at 55°C for 1 min and extension 72°C for 1 min, finishing with 1 cycle of final extension at 72°C for 10 min. For other 16 samples, where amplification with COH6/COL6b primers failed, another primer pair was used, LCO1490 (5’- GGT CAA CAA ATC ATA AAG ATA TTG G -3’) and HCO2198 (5’- TAA ACT TCA GGG TGA CCA AAA AAT CA -3’) [16], the thermal cycle included initial denaturation at 94°C for 1 min, 5 cycles of 94°C for 40 s, 45°C for 40 s, 72°C for 1 min, followed by 35 cycles of 94°C for 40 s, 51°C for 40 s, 72°C for 1 min and a final extension of 72°C for 5 min. For details on samples amplification primers, see S1 Table. The amplified products were visualized in 1% agarose gel, purified using Gel / PCR DNA fragment extraction kit (Geneaid) and sequenced with forward primer at Biology Section, Faculty of Science, Charles University using BigDye® Terminator v3.1 Cycle Sequencing Kit (Applied Biosystems) on POP-7 polymer (Applied Biosystems). Raw sequences were trimmed and edited in Geneious v10.2.6 (https://www.geneious.com) software and aligned by ClustalW approach [35] as implemented in Geneious. These sequence data have been submitted to the NCBI databases under accession numbers OR820652—OR820723 (see S1 Table).

Available DNA barcodes from samples collected in CAR (n = 73 [23]) were obtained from BOLD database. In addition, several unpublished sequences from Cameroon (n = 10) and Gabon (n = 10) were provided by Nicolas Moulin (Muséum national d’Histoire naturelle, Paris, France). Raw chromatograms were curated, converted to the sequences and aligned together with our sequences using same software tools as described above.

Pairwise genetic distances and maximum likelihood trees were calculated in IQ-TREE 1.6.12 [36]. Sequences were initially partitioned according to 1^st^, 2^nd^ and 3^rd^ codon position. ModelFinder [37] was used to determine the best partition scheme and nucleotide substitution model according to Bayesian information criterion. The best partition scheme was keeping the original three partitions, and the best models were TPM3+F+I+G4, TIM3e+I+G4 and GTR+F+G4, respectively. Support for the clades found on the ML tree was calculated using SH-aLRT, aBayes and ultrafast bootstrap [38].

A map of collecting sites (Fig 1) was created with open-source desktop geographic information system application QGIS 3.36 (QGIS Development Team) using Shuttle Radar Topography Mission (SRTM) digital elevation model [39]. Country boundaries and populated places data were obtained from https://www.naturalearthdata.com.

Abbreviations of institutions of type material deposition:

ANSP: Academy of Natural Sciences, Philadelphia, USA.

EMAU: Ernst-Moritz-Arndt-Universität Greifswald, Griefswald, Germany.

MNCN: Museo Nacional de Ciencias Naturales, Madrid, Spain.

MNHN: Muséum national d’Histoire naturelle, Paris, France.

MSNG: Museo Civico di Storia Naturale di Genova "Giacomo Doria", Genova, Italy.

MZFN: University of Naples Federico II, Naples, Italy.

MZH: Finnish Museum of Natural History, Helsinki, Finland.

NHMUK: The Natural History Museum, London, UK.

NHMW: Naturhistorisches Museum, Vienna, Austria.

NHRS: Naturhistoriska Riksmuseet, Stockholm, Sweden.

NZSI: Zoological Survey of India, Kolkata, India.

OUMNH: Oxford University Museum of Natural History, Oxford, UK.

RBINS: Institut Royal des Sciences Naturelles de Belgique, Bruxelles, Belgium.

RMCA: Musée Royal de l’Afrique Centrale, Tervuren, Belgium.

SMF: Forschungsinstitut und Natur-Museum Senckenberg, Frankfurt-am-Main, Germany.

ZMHB: Museum für Naturkunde der Humboldt-Universität, Berlin, Germany.

## Results

### Taxonomic checklist

**Fig 2:**
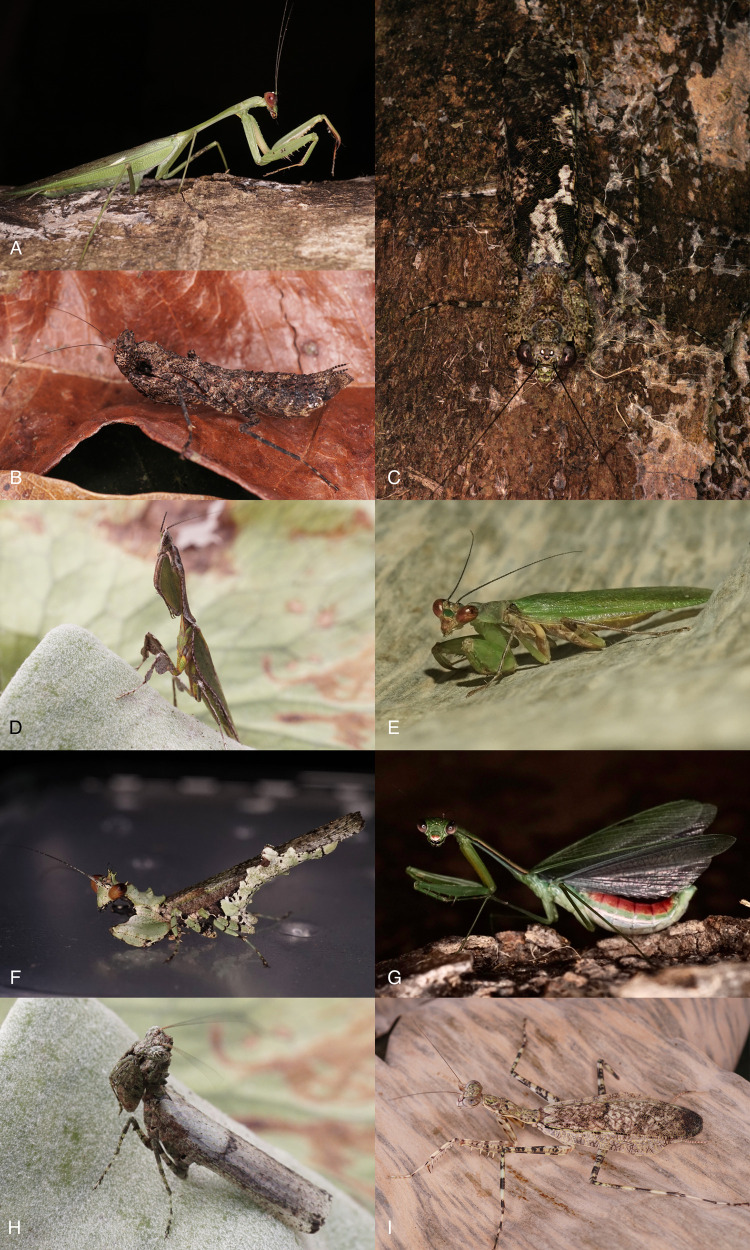
Live habitus of selected Mantodea collected in Ebogo II, Cameroon. A–*Tismomorpha vitripennis*; B–*Achlaena grandis*; C–*Theopompella aurivillii*; D–*Congoharpax aberrans*; E–*Panurgica rehni*; F–*Chrysomantis speciosa*; G–*Deromantis limbalicollis*; H–*Chrysomantis cachani*; I–*Dactylopteryx flexuosa*.

A total of 495 specimens (387 males, 108 females) were collected, representing 62 species belonging to 10 families and 35 genera of Mantodea. Eight species (*Amorphoscelis lamottei*, *Cataspilota cf*. *C*. *guineensis*, *Caudatoscelis caudata*, *Omomantis sigma*, *Prohierodula viridimarginata*, *Sphodromantis balachowskyi*, *Stenopyga ziela*, and *Tarachodes feae*), are reported for the first time for the country, while precise locations for *Panurgica rehni* and *Polyspilota pavani* improve previous brief records. At least five species (see taxonomic list below) are suspected to be new to science. Photographs of select live specimens are presented on Fig 2.

Family **Amorphoscelidae** Stål, 1877

*Amorphoscelis* Stål, 1871

*Amorphoscelis griffinii* Giglio-Tos, 1913

***Type locality*.** Punta Frailes, Fernando Po (= Bioko, Equatorial Guinea).

***Type deposition*.** Holotype ♂ MSNG.

***Material*.** 1♂ (VGPC23364): Mbalmayo Region, Ebogo II village, light trap 3°23’38.4"N 11°28’15.6"E, 6.VIII.2021, V. Govorov leg.

***Distribution*.** Cameroon, CAR, Democratic Republic of the Congo, Equatorial Guinea, Gabon, Ivory Coast, Rwanda.

*Amorphoscelis grisea* Bolivar, 1908

***Type locality*.** Cameroon.

***Type deposition*.** Holotype ♂ MNCN.

***Material*.** 2♂ (VGPC23365-6): Mbalmayo Region, Ebogo II village, light trap 3°23’38.4"N 11°28’15.6"E, 5-15.VIII.2021, V. Govorov leg.

***Distribution*.** Cameroon, CAR, Democratic Republic of the Congo, Gabon, Guinea, Ivory Coast, Republic of the Congo, Uganda.

*Amorphoscelis lamottei* Roy, 1963

***Type locality*.** Station Ziela, Mount Nimba, Guinea.

***Type deposition*.** Holotype ♂ MNHN.

***Material*.** 1♂ (VGPC23363): Mbalmayo Region, Ebogo II village, light trap 3°23’38.4"N 11°28’15.6"E, 10-11.VIII.2021, V. Govorov leg.

***Distribution*.** CAR, Democratic Republic of the Congo, Gabon, Ghana, Guinea, Ivory Coast, Tanzania, Republic of the Congo, Uganda.

***Comments*.** New record for Cameroon.

*Amorphoscelis cf*. *A*. *pulchra* Bolivar, 1908

***Material*.** 2♂ (VGPC23541-2): 1♂ Mbalmayo Region, Ebogo II village, light trap 3°23’38.4"N 11°28’15.6"E, 27.IV.2015, J. Šobotník leg.; 1♂, same data, but 26.I-9.III.2022.

***Comments*.** Two male specimens closely resemble *A*. *pulchra* but differ in genitalia structure from type specimen. More research is required.

*Bolivaroscelis* Roy, 1973

*Bolivaroscelis bolivarii* (Giglio-Tos, 1913)

***Type locality*.** Station Johann-Albrechtshöhe, Cameroon.

***Type deposition*.** Holotype ♀ ZMHB.

***Material*.** 1♂ (VGPC23544), Mbalmayo Region, Ebogo II village, light trap 3°23’38.4"N 11°28’15.6"E, 26.I-9.III.2022, J. Šobotník leg.

***Distribution*.** Cameroon, CAR, Democratic Republic of the Congo, Gabon, Republic of the Congo.

*Bolivaroscelis carinata* (Bolivar, 1908)

***Type locality*.** Cameroon.

***Type deposition*.** Holotype ♂ MNCN.

***Material*.** 1♀ nymph (VGPC23424), Mbalmayo Region, Ebogo II village, timber works 3°23’27.9"N 11°28’16.0"E, 22.VIII.2021, V. Govorov leg.

***Distribution*.** Cameroon, Gabon.

*Caudatoscelis* Roy, 1973

*Caudatoscelis caudata* (Giglio-Tos, 1914)

***Type locality*.** Republic of the Congo.

***Type deposition*.** Holotype ♂ MSNG.

***Material*.** 7♂, 12♀ (VGPC23351-8, VGPV23413-423): 5♂, 9♀, Mbalmayo Region, Ebogo II village, primary forest 3°23’01.6"N 11°27’50.7"E, 2-10.VIII.2021, V. Govorov leg.; 2♂, 3♀ same data, but 17-20.VIII.2021.

***Distribution*.** CAR, Gabon, Republic of the Congo.

***Comments*.** New record for Cameroon. Some immature specimens were observed to have a Torymidae wasp attached to its body.

*Gigliotoscelis* Roy, 1973

*Gigliotoscelis simulans* (Giglio-Tos, 1913)

***Type locality*.** ♂ Kratoki, Togo; ♀ Mukonje Farm near Mundame on the Mungo River, Cameroon.

***Type deposition*.** Syntype ♂ ZMHB.

***Material*.** 1♂ (VGPC23367): Mbalmayo Region, Ebogo II village, light trap 3°23’38.4"N 11°28’15.6"E, 15.VIII.2021, V. Govorov leg.

***Distribution*.** CAR, Cameroon, Gabon, Ghana, Guinea, Republic of the Congo, Togo.

*Maculatoscelis* Roy, 1973

*Maculatoscelis ascalaphoides* (Bolivar, 1908)

***Type locality*.** ‘Bonake Costa’, Cameroon.

***Type deposition*.** Holotype ♂ MNCN.

***Material*.** 5♂: 3♂, Mbalmayo Region, Ebogo II village, light trap 3°23’38.4"N 11°28’15.6"E, 30.VII-15.VIII.2021, V. Govorov leg.; 1♂, same data, but 1-31.III.2018, J. Synek leg.; 1♂, same data, but 26.I-9.III.2022, J. Šobotník leg.

***Distribution*.** Angola, Cameroon, CAR, Gabon, Ghana, Guinea, Republic of the Congo, Tanzania.

Family **Chroicopteridae** Giglio-Tos, 1915

*Achlaena* Karsch, 1892

*Achlaena grandis* (Westwood, 1889)

***Type locality*.** Fernando Po (Bioko, Equatorial Guinea).

***Type deposition*.** Holotype ♀ OUMNH.

***Material*.** 2♂, 2♀ (VGPC23331-2, VGPC23812-3): 1♀, Mbalmayo Region, Ebogo II village, primary forest 3°23’01.6"N 11°27’50.7"E, 31.VII.2021, V. Govorov leg.; 1♂, same data, but 1.VIII.2021; 1♂, same data, but 17.VIII.2021; 1♀, same data, but 18.VIII.2021.

***Distribution*.** Cameroon, Democratic Republic of the Congo, Equatorial Guinea, Ghana, Guinea, Ivory Coast.

***Comments*.** All specimens were collected by visual examination of vegetation in the forest, with males sitting on the underside of the leaves, and females resting on the ground or logs. One female was found to be infected with an unidentified larva of Tachinidae fly.

Family **Dactylopterygidae** Giglio-Tos, 1915

*Dactylopteryx* Karsch, 1892

*Dactylopteryx flexuosa* Karsch, 1892

***Type locality*.** Gabon.

***Type deposition*.** Holotype ♀ ZMHB.

***Material*.** 2♂, 2♀ (VGPC23394-5, VGPC23456, VGPC23545): 1♂, 1♀ Mbalmayo Region, Ebogo II village, primary forest 3°23’01.6"N 11°27’50.7"E, 7.VIII.2021, V. Govorov leg.; 1♂, 1♀ nymphs, same data, but 2-10.VIII.2021.

***Distribution*.** Cameroon, CAR, Congo, Gabon, Ghana, Ivory Coast.

*Theopompella* Giglio-Tos, 1917

*Theopompella aurivillii* (Sjöstedt, 1900)

***Type locality*.** Mapanga, Cameroon.

***Type deposition*.** Holotype ♀ NHRS.

***Material*.** 2♂ (VGPC23387-7): 1♂, Mbalmayo Region, Ebogo II village, light trap 3°23’38.4"N 11°28’15.6"E, 5.VIII.2021, V. Govorov leg.; 1♂, same data, but 13.VIII.2021.

***Distribution*.** Angola, Cameroon, CAR, Democratic Republic of the Congo, Equatorial Guinea, Gabon, Malawi, Republic of the Congo.

*Theopompella heterochroa* (Gerstaecker, 1883)

***Type locality*.** Quaqua, Cameroon.

***Type deposition*.** Holotype ♂ EMAU.

***Material*.** 3♂ (VGPC23388, VGPC23579-80): 2♂, Mbalmayo Region, Ebogo II village, light trap 3°23’38.4"N 11°28’15.6"E, 25.V-2.VI.2021, J. Šobotník leg.; 1♂ same data, but 22.VIII.2021, V. Govorov leg.

***Distribution*.** Cameroon, CAR, Democratic Republic of the Congo, Gabon, Kenya, Nigeria.

Family **Deroplatyidae** Westwood, 1889

*Leptocola* Gerstaecker, 1883

*Leptocola stanleyana* (Westwood, 1889)

***Type locality*.** Gabon.

***Type deposition*.** Holotype ♀ OUMNH.

***Material*.** 2♀ nymphs (VGPC23403, VGPC23462): Mbalmayo Region, Ebogo II village, primary forest 3°23’01.6"N 11°27’50.7"E, 18.VIII.2021, V. Govorov leg.

***Distribution*.** Democratic Republic of the Congo, Gabon, Cameroon, Mozambique, Nigeria, Tanzania, Togo.

*Macrodanuria* Sjöstedt, 1900

*Macrodanuria elongata* (Borre de Preudhomme, 1883)

***Type locality*.** Conde, Guinea.

***Type deposition*.** Holotype ♂ RBINS.

***Material*.** 2♂ (VGPC23581-2): Mbalmayo Region, Ebogo II village, light trap 3°23’38.4"N 11°28’15.6"E, 26.I-9.III.2022, J. Šobotník leg.

***Distribution*.** Cameroon, CAR, Gabon, Ghana, Guinea, Ivory Coast.

*Stenopyga* Karsch, 1892

*Stenopyga (Stenopyga) extera* Karsch, 1892

***Type locality*.** Lake Barombi, Cameroon.

***Type deposition*.** Holotype ♂♀ ZMHB.

***Material*.** 2♂, 1♀ (VGPC23383, VGPC23532-3): 1♂ Mbalmayo Region, Ebogo II village, light trap 3°23’38.4"N 11°28’15.6"E, 27.VII-1.VIII.2021, V. Govorov leg.; 1♀ nymph same data, but 22.VIII.2021; 1♂ same data, but, 25.V-2.VI.2021, J. Šobotník leg.

***Distribution*.** Cameroon, Gabon, Liberia, Republic of the Congo.

*Stenopyga (Stenopyga) ziela* Roy, 1963

***Type locality*.** Station Ziela, Mount Nimba, Guinea.

***Type deposition*.** Holotype ♂ MNHN.

***Material*.** 4♂ (VGPC23382, VGPC23534-6): 4♂ Mbalmayo Region, Ebogo II village, light trap 3°23’38.4"N 11°28’15.6"E, 11-22.VIII.2021, V. Govorov leg.

***Distribution*.** CAR, Democratic Republic of the Congo, Gabon, Ghana, Guinea, Ivory Coast, Republic of the Congo.

***Comments*.** New record for Cameroon.

Family **Eremiaphilidae** Saussure, 1869

***Galepsus*** Stål, 1876

*Galepsus* sp.

***Material*.** 1♂ (VGPC23806): Mbalmayo Region, Ebogo II village, primary forest 3°23’01.6"N 11°27’50.7"E, 18.VIII.2021, V. Govorov leg.

***Comments*.** Collected unique male specimen probably belongs to an undescribed taxon and is the subject of an upcoming publication.

*Tarachodes* Giglio-Tos, 1911

*Tarachodes (Tarachodes) feae* Giglio-Tos, 1911

***Type locality*.** Fernando Vaz (= Nkomi Lagoon), Gabon.

***Type deposition*.** Holotype ♂ MNCN.

***Material*.** 2♂ (VGPC23389-90): Mbalmayo Region, Ebogo II village, light trap 3°23’38.4"N 11°28’15.6"E, 7-15.VIII.2021, V. Govorov leg.

***Distribution*.** Gabon, Republic of the Congo.

***Comments*.** New record for Cameroon.

*Tarachodes* (*Tarachodina*) *gerstaeckeri* Werner, 1907

***Type locality*.** Banyana, Cameroon.

***Type deposition*.** Holotype ♂ ZMHB.

***Material*.** 1♂ (VGPC23539): Mbalmayo Region, Ebogo II village, light trap 3°23’38.4"N 11°28’15.6"E, 26.I-9.III.2022, J. Šobotník leg.

***Distribution*.** Cameroon, CAR, Gabon, Ghana, Guinea, Ivory Coast, Togo.

Family **Galinthiadidae** Giglio-Tos, 1919

*Congoharpax* La Greca, 1954

*Congoharpax aberrans* La Greca, 1954

***Type locality*.** Democratic Republic of the Congo.

***Type deposition*.** Holotype ♂ RMCA.

***Material*.** 1♂ (VGPC23375): Mbalmayo Region, Ebogo II village, light trap 3°23’38.4"N 11°28’15.6"E, 15.VIII.2021, V. Govorov leg.

***Distribution*.** Cameroon, CAR, Democratic Republic of the Congo, Gabon, Ghana, Guinea, Ivory Coast, Republic of the Congo, Togo.

Family **Hymenopodidae** Giglio-Tos, 1915

*Anasigerpes* Giglio-Tos, 1915

*Anasigerpes bifasciata* Giglio-Tos, 1915

***Type locality*.** Estudan-Manf, signal Bascho, Cameroon.

***Type deposition*.** Syntypes ♂, 2♀ ZMHB.

***Material*.** 10♂, 2♀ (VGPC23368-70, VGPC23426-34): 2♂ Mbalmayo Region, Ebogo II village, light trap 3°23’38.4"N 11°28’15.6"E, 27.VII-1.VIII.2021, V. Govorov leg.; 4♂ same data, but 2-10.VIII.2021; 1♂, 1♀ same data, but 14.VIII.2021; 3♂ same data, but 26.I-9.III.2022, J. Šobotník leg.; 1♀ same data, but 1-31.III.2018, J. Synek leg.

***Distribution*.** Angola, Cameroon, CAR, Democratic Republic of the Congo, Equatorial Guinea, Gabon, Ghana, Guinea, Ivory Coast, Nigeria, Republic of the Congo, Uganda.

*Anasigerpes heydeni* (Werner, 1908)

***Type locality*.** Unknown.

***Type deposition*.** Syntypes ♀ SMF.

***Material*.** 1♂ (VGPC23435): Mbalmayo Region, Ebogo II village, light trap 3°23’38.4"N 11°28’15.6"E, 26.I-9.III.2022, J. Šobotník leg.

***Distribution*.** Cameroon, CAR, Democratic Republic of the Congo, Gabon, Guinea, Ivory Coast, Kenya, Republic of the Congo, Uganda.

*Chlidonoptera* Karsch, 1892

*Chlidonoptera vexillum* Karsch, 1892

***Type locality*.** Buea, Cameroon.

***Type deposition*.** Holotype ♀ ZMHB.

***Material*.** 15♂ (VGPC23342-3, VGPC23506-18): 1♂ Mbalmayo Region, Ebogo II village, light trap 3°23’38.4"N 11°28’15.6"E, 27.VII-1.VIII.2021, V. Govorov leg.; 5♂ same data, but 11-22.VIII.2021; 2♂ same data, but 25.V-2.VI.2021, J. Šobotník leg.; 7♂ same data, but 26.I-9.III.2022, J. Šobotník leg.

***Distribution*.** Cameroon, CAR, Democratic Republic of the Congo, Gabon, Republic of the Congo, Tanzania, Uganda.

*Chloroharpax* Werner, 1908

*Chloroharpax modesta* (Gerstaecker, 1883)

***Type locality*.** Cameroon.

***Type deposition*.** Holotype ♂ EMAU.

***Material*.** 8♂ (VGPC23381, VGPC23442-8): 6♂ Mbalmayo Region, Ebogo II village, light trap 3°23’38.4"N 11°28’15.6"E, 2-10.VIII.2021, V. Govorov leg.; 1♂ same data, but 11-23.VIII.2021; 1♂ same data, but 26.I-9.III.2022, J. Šobotník leg.

***Distribution*.** Cameroon, CAR, Democratic Republic of Congo, Gabon, Ghana, Guinea, Ivory Coast, Nigeria, Republic of the Congo.

*Chrysomantis* Giglio-Tos, 1915

*Chrysomantis cachani* (Roy, 1964)

***Type locality*.** Grabo, Ivory Coast.

***Type deposition*.** Holotype ♂ MNHN.

***Material*.** 2♂ (VGPC23379-80): 1♂ Mbalmayo Region, Ebogo II village, light trap 3°23’38.4"N 11°28’15.6"E, 14-5.VIII.2021, V. Govorov leg.

***Distribution*.** CAR, Cameroon, Democratic Republic of Congo, Gabon, Ghana, Guinea, Ivory Coast, Republic of the Congo, Tanzania.

*Chrysomantis speciosa* Giglio-Tos, 1915

***Type locality*.** Aburi, Ghana.

***Type deposition*.** Holotype ♂ NHMUK.

***Material*.** 2♂ (VGPC23378, VGPC23540): 1♂ Mbalmayo Region, Ebogo II village, light trap 3°23’38.4"N 11°28’15.6"E, 31.VII.2021, V. Govorov leg.; 1♂ same data, but 26.I-9.III.2022, J. Šobotník leg.

***Distribution*.** Angola, CAR, Cameroon, Democratic Republic of Congo, Gabon, Ghana, Ivory Coast, Republic of the Congo, Uganda.

*Oxypiloidea* Schulthess, 1898

*Oxypiloidea (Catasigerpes) camerunensis* (Giglio-Tos, 1915)

***Type locality*.** Lolodorf, Cameroon.

***Type deposition*.** Holotype ♂ ZMHB.

***Material*.** 1♂ (VGPC23376): Mbalmayo Region, Ebogo II village, light trap 3°23’38.4"N 11°28’15.6"E, 10-11.VIII.2021, V. Govorov leg.

***Distribution*.** Cameroon, Gabon, Republic of the Congo.

*Oxypiloidea (Catasigerpes) margarethae* (Werner, 1912)

***Type locality*.** Diré Doûa, Ethiopia.

***Type deposition*.** Holotype ♂ NHMW.

***Material*.** 6♂ (VGPC23377, VGPC23459-60, VGPC23495-7): 4♂, Mbalmayo Region, Ebogo II village, light trap 3°23’38.4"N 11°28’15.6"E, 27.VII-22.VIII.2021, V. Govorov leg.; 2♂, same data, but 26.I-9.III.2022, J. Šobotník leg.

***Distribution*.** Cameroon, CAR, Chad, Democratic Republic of the Congo, Eritrea, Ethiopia, Gabon, Kenya, Niger, Nigeria, Sudan, Uganda.

*Panurgica* Karsch, 1896

*Panurgica feae* Griffini, 1907

***Type locality*.** Fea Basile, Fernando Po (Bioko, Equatorial Guinea).

***Type deposition*.** Holotype ♀ MSNG.

***Material*.** 6♂ (VGPC23371-3, VGPC23498-500): 2♂ Mbalmayo Region, Ebogo II village, light trap 3°23’38.4"N 11°28’15.6"E, 31.VII.2021, V. Govorov leg.; 1♂ same data, but 16-22.VIII.2021; 3♂ same data, but 26.I-9.III.2022, J. Šobotník leg.

***Distribution*.** Angola, Cameroon, CAR, Equatorial Guinea, Gabon, Republic of the Congo.

*Panurgica rehni* (La Greca, 1954)

***Type locality*.** Maniema Kindu, Tshuapa Eala, Libangi Karawa, Democratic Republic of Congo.

***Type deposition*.** Holotype ♂ RMCA.

***Material*.** 1♂ (VGPC23374): Mbalmayo Region, Ebogo II village, light trap 3°23’38.4"N 11°28’15.6"E, 11.VIII.2021, V. Govorov leg.

***Distribution*.** Cameroon, CAR, Democratic Republic of Congo, Gabon, Republic of the Congo.

***Comments*.** Roy [24] mentions this species being present in Cameroon without providing any specified locality data. This is the first precise record of this species for the country.

*Phyllocrania* Burmeister, 1838

*Phyllocrania paradoxa* Burmeister, 1838

***Type locality*.** Cape Province (South Africa).

***Type deposition*.** Holotype ♀ ZMHB.

***Material*.** 2♂ (VGPC23577-8): Mbalmayo Region, Ebogo II village, light trap 3°23’38.4"N 11°28’15.6"E, 26.I-9.III.2022, J. Šobotník leg.

***Distribution*.** Angola, Cameroon, CAR, Democratic Republic of the Congo, Ethiopia, Gabon, Ghana, Guinea, Ivory Coast, Kenya, Madagascar, Mozambique, Namibia, Somalia, South Africa, Sudan, Tanzania, Togo, Uganda, Zimbabwe.

*Sibylla* Stål, 1877

*Sibylla (Sibylla) dolosa* Roy, 1975

***Type locality*.** La Maboké, CAR.

***Type deposition*.** Holotype ♂ MNHN.

***Material*.** 1♂, 2♀ (VGPC23399-400, VGPC23463): 1♀ nymph, Mbalmayo Region, Ebogo II village, primary forest 3°23’01.6"N 11°27’50.7"E, 1.VIII.2021, V. Govorov leg.; 1♀ nymph, same data, but 14.VIII.2021; 1♂ nymph, same data, but 21.VIII.2021.

***Distribution*.** Angola, Cameroon, CAR, Democratic Republic of the Congo, Gabon, Gambia, Ghana, Guinea, Ivory Coast, Nigeria.

***Comments*.** Specimens were found exclusively on tree trunks densely covered in epiphytes, always no lower than 2 m above ground.

*Sibylla (Sibyllopsis) griffinii* Giglio-Tos, 1915

***Type locality*.** Nzerekore, Guinea.

***Type deposition*.** Holotype ♂ MZH.

***Material*.** 1♂ (VGPC23588): Mbalmayo Region, Ebogo II village, light trap 3°23’38.4"N 11°28’15.6"E, 25.V-2.VI.2022, J. Šobotník leg.

***Distribution*.** Benin, Cameroon, CAR, Democratic Republic of Congo, Equatorial Guinea, Gabon, Ghana, Guinea, Ivory Coast, Liberia, Nigeria, Sierra Leone, Togo.

*Sibylla (Sibyllopsis) pannulata* Karsch, 1894

***Type locality*.** Buea, Cameroon.

***Type deposition*.** Holotype ♂ ZMHB.

***Material*.** 15♂, 2♀ (VGPC23394-8, VGPC23594-605): 1♂, 1♀ Mbalmayo Region, Ebogo II village, light trap 3°23’38.4"N 11°28’15.6"E, 27.VII-1.VIII.2021, V. Govorov leg.; 8♂ same data, but 2-10.VIII.2021; 5♂ same data, but 26.I-9.III.2022, J. Šobotník leg.; 1♂ same data, but 15.VIII-1.IX.2017, J. Synek leg.; 1♀ same data, but 1-31.III.2018, J. Synek leg.

***Distribution*.** Angola, Cameroon, CAR, Democratic Republic of the Congo, Equatorial Guinea, Gabon, Nigeria, Republic of the Congo.

*Sibylla (Sibyllopsis) vanderplaetseni* Roy, 1963

***Type locality*.** Station Ziela, Mount Nimba, Guinea.

***Type deposition*.** Holotype ♂ MNHN.

***Material*.** 7♂, 1♀(VGPC23391-3, VGPC23589-93): 3♂ Mbalmayo Region, Ebogo II village, light trap 3°23’38.4"N 11°28’15.6"E, 30.VII-10.VIII.2021, V. Govorov leg.; 3♂ same data, but 26.I-9.III.2022, J. Šobotník leg.; 1♀ same data, but 18.VIII-1.IX.2017, J. Synek leg.; 1♂ same data, but 18.III-18.IV.2018, J. Synek leg.

***Distribution*.** Angola, Cameroon, CAR, Democratic Republic of the Congo, Gabon, Ghana, Guinea, Ivory Coast, Republic of the Congo, Uganda.

Family **Mantidae** Latereille, 1802

*Alalomantis* Giglio-Tos, 1917

*Alalomantis muta* (Wood-Mason, 1882)

***Type locality*.** Buea, Cameroon.

***Type deposition*.** Holotype ♀ NZSI.

***Material*.** 34♂, 11♀ (VGPC23336-9, VGPC23449-53, VGPC23489-90, VGPC23): 20♂, 5♀ Mbalmayo Region, Ebogo II village, light trap 3°23’38.4"N 11°28’15.6"E, 27.VII-1.VIII.2021, V. Govorov leg.; 6♂, 3♀ same data, but 2-10.VIII.2021; 2♂ same data, but 11-22.VIII.2021; 1♂ same data, but 26.I-9.III.2022, J. Šobotník leg; 4♂, 2♀ same data, but 25.V-2.VI.2021, J. Šobotník leg.; 1♂, 1♀ same data, but 1-31.III.2018, J. Synek leg.

***Distribution*.** Angola, Cameroon, CAR, Democratic Republic of the Congo, Equatorial Guinea, Gabon, Uganda.

***Comments*.** One collected adult male was found with a Torymidae wasp attached to its body.

*Cataspilota* Giglio-Tos, 1917

*Cataspilota calabarica* Westwood, 1889

***Type locality*.** Calabar, Nigeria.

***Type deposition*.** Holotype ♂ OUMNH.

***Material*.** 9♂, 1♀ (VGPC23314, VGPC23316, VGPC23696-703): 2♂ Mbalmayo Region, Ebogo II village, light trap 3°23’38.4"N 11°28’15.6"E, 27.VII-1.VIII.2021, V. Govorov leg.; 2♂, 1♀: same data, but 2-10.VIII.2021; 3♂ same data, but 11-22.VIII.2021; 2♂ same data, but 26.I-9.III.2022, J. Šobotník leg.

***Distribution*.** Cameroon, CAR, Equatorial Guinea, Gabon, Liberia, Nigeria.

*Cataspilota lolodorfana* (Giglio-Tos, 1911)

***Type locality*.** Lolodorf, Cameroon.

***Type deposition*.** Holotype ♂ ZMHB.

***Material*.** 14♂ (VGPC23682-95): 9♂ Mbalmayo Region, Ebogo II village, light trap 3°23’38.4"N 11°28’15.6"E, 2-10.VIII.2021, V. Govorov leg.; 4♂ same data, but 26.I-9.III.2022, J. Šobotník leg.; 1♂ same data, but 25.V-2.VI.2021, J. Šobotník leg.

***Distribution*.** Cameroon, CAR Democratic Republic of the Congo, Gabon, Nigeria, Republic of the Congo.

***Comments*.** One collected adult male was found with a Torymidae wasp attached to its body.

*Cataspilota tristis* (Giglio-Tos, 1911)

***Type locality*.** Ngoko station, Cameroon.

***Type deposition*.** Holotype ♂ ZMHB.

***Material*.** 4♂, 1♀ (VGPC23326-8, VGPC23681, VGPC23814): 1♀ Mbalmayo Region, Ebogo II village, primary forest 3°23’01.6"N 11°27’50.7"E, 28.VII.2021; 2♂ same data, but 2.VIII.2021, V. Govorov leg.; 1♂ same data, but 26.I-9.III.2022, J. Šobotník leg.; 1♂ same data, but 1-31.III.2018, J. Synek leg.

***Distribution*.** Cameroon, CAR, Gabon.

*Cataspilota* cf. *C*. *guineensis* (Giglio-Tos, 1917)

***Type locality*.** Guinea.

***Type deposition*.** Holotype ♀ ZMHB.

***Material*.** 3♂, 3♀ (VGPC23311-3, VGPC23315, VGPC23324-5): Mbalmayo Region, Ebogo II village, primary forest 3°23’01.6"N 11°27’50.7"E, 31.VII-2.VIII.2021, V. Govorov leg.

***Distribution*.** Gabon, Guinea.

***Comments*.** The genus is in need of revision, identification to species level is not reliable.

*Deromantis* Giglio-Tos, 1916

*Deromantis limbalicollis* (Karsch, 1892)

***Type locality*.** Kribi, Cameroon.

***Type deposition*.** Holotype ♂ ZMHB.

***Material*.** 1♂ (VGPC23537): Mbalmayo Region, Ebogo II village, light trap 3°23’38.4"N 11°28’15.6"E, 10-11.VIII.2021, V. Govorov leg.

***Distribution*.** Cameroon, CAR, Democratic Republic of Congo, Gabon, Republic of the Congo.

*Omomantis* Saussure, 1899

*Omomantis sigma* Rehn, 1949

***Type locality*.** Lukolela, Democratic Republic of the Congo.

***Type deposition*.** Holotype ♂ ANSP.

***Material*.** 7♂ (VGPC2340-1, VGPC23583-7): 3♂ Mbalmayo Region, Ebogo II village, light trap 3°23’38.4"N 11°28’15.6"E, 2-10.VIII.2021, V. Govorov leg.; 3♂ same data, but 26.I-9.III.2022, J. Šobotník leg.; 1♂ same data, but 1-31.III.2018, J. Synek leg.

***Distribution*.** CAR, Democratic Republic of the Congo, Gabon, Republic of the Congo.

***Comments*.** New record for Cameroon.

*Plistospilota* Giglio-Tos, 1911

*Plistospilota* cf. *P*. *maxima* Giglio-Tos, 1917

***Type locality*.** Unknown.

***Type deposition*.** Holotype ♂ NHMUK?

***Material*.** 29♂ (VGPC23321-3, VGPC23704-29): 1♂ Mbalmayo Region, Ebogo II village, light trap 3°23’38.4"N 11°28’15.6"E, 27.VII-1.VIII.2021, V. Govorov leg.; 5♂ same data, but 2-10.VIII.2021; 9♂ same data, but 11-22.VIII.2021; 12♂ same data, but 26.I-9.III.2022.2021, J. Šobotník leg; 2♂ same data, but 25.V-2.VI.2022, J. Šobotník leg.

***Distribution*.** Cameroon, CAR, Democratic Republic of the Congo, Ivory Coast?, Gabon, Republic of the Congo.

***Comments*.** The genus is in urgent need of revision, identification to species level is not reliable.

*Plistospilota* cf. *P*. *validissima* (Gerstaecker, 1883)

***Type locality*.** Aburi, Ghana.

***Type deposition*.** Holotype ♂ EMAU.

***Material*.** 4♂ (VGPC23730-3): 2♂ Mbalmayo Region, Ebogo II village, light trap 3°23’38.4"N 11°28’15.6"E, 27.VII-1.VIII.2021, V. Govorov leg.; 2♂ same data, but 26.I-9.III.2022.2021, J. Šobotník leg.

***Distribution*.** Cameroon, CAR, Democratic Republic of the Congo, Gabon, Ghana, Ivory Coast, Liberia.

***Comments*.** The genus is in urgent need of revision, identification to species level is not reliable.

*Polyspilota* Burmeister, 1838

*Polyspilota aeruginosa* (Goeze, 1778)

***Type locality*.** Unknown.

***Type deposition*.** Holotype? EMAU.

***Material*.** 27♂, 25♀ (VGPC2353-5, VGPC23519-31, VGPC23735-70): 7♂, 10♀ Mbalmayo Region, Ebogo II village, light trap 3°23’38.4"N 11°28’15.6"E, 27.VII-1.VIII.2021, V. Govorov leg.; 7♂, 6♀ same data, but 2-10.VIII.2021; 9♂, 3♀ same data, but 11-22.VIII.2021; 3♂, 3♀ same data, but 26.I-9.III.2022.2021, J. Šobotník leg; 1♂, 3♀ same data, but 1.VI.2022, J. Šobotník leg.

***Distribution*.** Angola, Cameroon, Cape Verde, CAR, Comoros, Democratic Republic of the Congo, Ethiopia, Gabon, Ghana, Guinea, Kenya, Liberia, Madagascar, Namibia, Republic of the Congo, South Africa, Tanzania, Uganda, Zanzibar, Zimbabwe.

***Comments*.** One adult male was collected with a Torymidae wasp attached under the wings, while another immature female was found to be infected with Tachinidae larva.

*Polyspilota pavani* La Greca, 1966

***Type locality*.** La Maboké, CAR.

***Type deposition*.** Holotype ♂ RMCA.

***Material*.** 3♂ (VGPC23329-30, VGPC23734): 2♂ Mbalmayo Region, Ebogo II village, light trap 3°23’38.4"N 11°28’15.6"E, 30.VII-12.VIII.2021, V. Govorov leg.; 1♂ same data, but 26.I-9.III.2022.2021, J. Šobotník leg.

***Distribution*.** Cameroon, CAR, Gabon.

***Comments*.** Roy [24] mentions this species being present in Cameroon without providing any specified locality data. This is the first precise record of this species for the country.

*Prohierodula* Bolivar, 1908

*Prohierodula laticollis* (Karsch, 1892)

***Type locality*.** Lake Barombi, Cameroon.

***Type deposition*.** Holotype ♂ ZMHB.

***Material*.** 62♂ (VGPC23319-20, VGPC23621-80,): 12♂ Mbalmayo Region, Ebogo II village, light trap 3°23’38.4"N 11°28’15.6"E, 27.VII-1.VIII.2021, V. Govorov leg.; 23♂ same data, but 2-10.VIII.2021; 9♂ same data, but 11-22.VIII.2021; 15♂ same data, but 26.I-9.III.2022, J. Šobotník leg; 2♂ same data, but 25.V-2.VI.2021, J. Šobotník leg.; 1♂ same data, but 1-31.III.2018, J. Synek leg.

***Distribution*.** Cameroon, CAR, Democratic Republic of the Congo, Gabon.

***Comments*.** One adult male was found to be infected by a horsehair worm (*Chordodes* sp., Nematomorpha).

*Prohierodula mundamensis* Giglio-Tos, 1911

***Type locality*.** Mukonje Farm near Mundame on the Mungo River, Cameroon.

***Type deposition*.** Holotype ♂ ZMHB.

***Material*.** 1♂ (VGPC23538): Mbalmayo Region, Ebogo II village, light trap 3°23’38.4"N 11°28’15.6"E, 25.V-2.VI.2021, J. Šobotník leg.

***Distribution*.** Cameroon, CAR, Gabon, Republic of the Congo.

***Comments*.** DNA sequence obtained from this species does not cluster together with other species of *Prohierodula*, rather than branches with *Cataspilota* and *Polyspilota*. This might indicate the currently unresolved relationships within tribe *Polyspilotini*.

*Prohierodula picta* (Gerstaecker, 1883)

***Type locality*.** Victoria (= Limbé), Cameroon.

***Type deposition*.** Holotype ♂ EMAU.

***Material*.** 12♂, 5♀ (VGPC23317-8, VGPC23457-8, VGPC23493-4, VGPC23606-16): 6♂, 3♀ Mbalmayo Region, Ebogo II village, light trap 3°23’38.4"N 11°28’15.6"E, 27.VII-1.VIII.2021, V. Govorov leg.; 4♂, 2♀ same data, but 2-10.VIII.2021; 2♂ same data, but 11-22.VIII.2021.

***Distribution*.** Cameroon, CAR, Equatorial Guinea, Gabon.

***Comments*.** Adult females were observed resting on the underside of plant leaves, guarding the ootheca.

*Prohierodula viridimarginata* La Greca, 1956

***Type locality*.** Epulu, Kibali Ituri, Uele Ibembo, Democratic Republic of the Congo.

***Type deposition*.** Holotype ♂ MZFN.

***Material*.** 5♂ (VGPC23488, VGPC23617-20): 1♂ Mbalmayo Region, Ebogo II village, light trap 3°23’38.4"N 11°28’15.6"E, 27.VII-1.VIII.2021, V. Govorov leg.; 2♂ same data, but 2-10.VIII.2021; 1♂ same data, but 11-22.VIII.2021; 1♂ same data, but 25.V-2.VI.2021, J. Šobotník leg.

***Distribution*.** CAR, Democratic Republic of the Congo, Gabon.

***Comments*.** New record for Cameroon.

*Sphodromantis* Stål, 1871

*Sphodromantis aureoides* Roy, 2010

***Type locality*.** Mundame, Cameroon.

***Type deposition*.** Holotype ♂ NHMW.

***Material*.** 2♂, 2♀ (VGPC23815-8): South-West Region, Limbé, secondary forest, 1-30.VIII.2019, Ntah Eliot Nji Bama leg.

***Distribution*.** Cameroon.

***Comments*.** Roy [40] suggested, judging by the absence of this species in collection records from 1970 onwards, that *S*. *aureoides* might be a victim of aggressive competition from dominant *Sphodromants lineola pinguis* La Greca, 1967, and possibly, hybridisation with it, therefore disappearing from its distribution area. Our findings show that this remarkable species continues to exist in Cameroon.

*Sphodromantis balachowskyi* La Greca, 1967

***Type locality*.** Makokou, Gabon.

***Type deposition*.** Holotype ♂ coll. Pavan: CAR.

***Material*.** 3♂ (VGPC23402, VGPC23572-3): 1♂ Mbalmayo Region, Ebogo II village, light trap 3°23’38.4"N 11°28’15.6"E, 8.VIII.2021, V. Govorov leg.; 2♂ same data, but 26.I-9.III.2022, J. Šobotník leg.

***Distribution*.** CAR, Democratic Republic of the Congo, Gabon.

***Comments*.** New record for Cameroon.

*Sphodromantis gracilicollis centroccidentalis* Roy, 2010

***Type locality*.** La Maboké, CAR.

***Type deposition*.** Holotype ♂ MNHN.

***Material*.** 1♂ (VGPC23574): Mbalmayo Region, Ebogo II village, light trap 3°23’38.4"N 11°28’15.6"E, 26.I-9.III.2022, J. Šobotník leg.

***Distribution*.** Cameroon, CAR, Gabon.

*Sphodromantis lineola pinguis* La Greca, 1967

***Type locality*.** La Maboké, CAR.

***Type deposition*.** Holotype ♂ RMCA.

***Material*.** 27♂, 7♀ (VGPC23401, VGPC23440-1, VGPC23501-5, VGPC23546-71): 4♂, 1♀ Mbalmayo Region, Ebogo II village, light trap 3°23’38.4"N 11°28’15.6"E, 27.VII-1.VIII.2021, V. Govorov leg.; 9♂, 3♀ same data, but 2-10.VIII.2021; 9♂, 1♀ same data, but 11-22.VIII.2021; 3♂, 1♀ same data, but 26.I-9.III.2022, J. Šobotník leg; 1♂ same data, but 25.V-2.VI.2021, J. Šobotník leg.; 1♂ same data, but 1-31.III.2018, J. Synek leg; 1♀ Mbalmayo Region, Ebogo II village, bank of the Nyong River 3°25’03.8"N 11°27’12.8"E, 10.VIII.2021, V. Govorov leg.

***Distribution*.** Angola, CAR, Cameroon, Democratic Republic of the Congo, Gabon, Republic of the Congo.

***Comments*.** This species was extremely abundant in disturbed habitats (village, plantations), but noticeably absent in the forest. One female (10.VIII.2021) was found resting on the stand of reeds along Nyong River right above moving water. Such an unusual place for a mantis was most likely linked to this individual bearing two horsehair worms (*Chordodes* sp., Nematomorpha) which are known to drive its hosts towards open water to continue the parasite’s lifecycle.

*Sphodromantis* sp.

***Material*.** 1♂, 1♀ (VGPC23): South-West Region, Limbé, secondary forest, 1-30.VIII.2019, Ntah Eliot Nji Bama leg.

***Comments*.** Closely allied to *S*. *lineola pinguis* but differ in body size and presence of pigmented spots on the forefemora. See Discussion for more details.

*Tenodera* Burmeister, 1838

*Tenodera superstitiosa superstitiosa* (Fabricius, 1781)

***Type locality*.** Equatorial Africa.

***Type deposition*.** Holotype ♀ NHMUK.

***Material*.** 1♂ (VGPC23576): Mbalmayo Region, Ebogo II village, around light trap 3°23’38.4"N 11°28’15.6"E, 1.VI.2021, J. Šobotník leg.

***Distribution*.** Angola, Cameroon, Democratic Republic of the Congo, Ethiopia, Gabon, Ghana, Guinea, Kenya, Liberia, Malawi, Mozambique, Namibia, Nigeria, Senegal, Somalia, South Africa, Sudan, Tanzania, Togo, Uganda, Zambia.

*Tismomorpha* Roy, 1973

*Tismomorpha vitripennis* (Bolivar, 1908)

***Type locality*.** Esosung, Bakossi Mountains, Cameroon.

***Type deposition*.** Holotype ♂ MNCN.

***Material*.** 4♂ (VGPC23333-5, VGPC23575): 1♂ Mbalmayo Region, Ebogo II village, light trap 3°23’38.4"N 11°28’15.6"E, 30.VII.2021, V. Govorov leg.; 1♂ same data, but 5.VIII.202; 1♂ same data, but 26.I-9.III.2022, J. Šobotník leg.; 1♂ same data, but 1-31.III.2018, J. Synek leg.

***Distribution*.** Cameroon, CAR, Gabon, Republic of the Congo.

Family **Miomantidae** Westwood, 1889

*Miomantis* Saussure, 1870

*Miomantis preussi* Karsch, 1892

***Type locality*.** Lake Barombi, Cameroon.

***Type deposition*.** Holotype 2♂ ZMHB.

***Material*.** 8♂, 15♀ (VGPC23436-9, VGPC23469-87): 4♀, 4♂ Mbalmayo Region, Ebogo II village, primary forest 3°23’01.6"N 11°27’50.7"E, 27.VII-1.VIII.2021, V. Govorov leg.; 8♀, 3♂ same data, but 2-10.VIII.2021; 3♀, 1♂ same data, but 11-22.VIII.2021.

***Distribution*.** Cameroon, CAR, Equatorial Guinea (Bioko Island), Gabon.

***Comments*.** Adult females were observed resting on the underside of plant leaves, guarding the ootheca.

Family **Nanomantidae** Brunner de Wattenwyl, 1893

*Hapalomantis* Saussure, 1871

*Hapalomantis (Bolbira)* sp.

***Material*.** 1♂ (VGPC23811): Mbalmayo Region, Ebogo II village, light trap 3°23’38.4"N 11°28’15.6"E, 4.VIII.2021, V. Govorov leg.

***Comments*.** Comparing unique male collected in Ebogo II with other species in genus, it closely resembles *Hapalomantis (Bolbira) minima* (Werner, 1906), however, the latter species is distributed in Eastern and South Africa. Type specimen is reported by Ehrmann [30] to be deposited in the State Museum of Natural History in Stuttgart but could not be located there. More research is required for a precise identification of this specimen.

*Negromantis* Giglio-Tos, 1915

*Negromantis lutescens* (Sjöstedt, 1900)

***Type locality*.** Kitta, Cameroon.

***Type deposition*.** Holotype ♂ NHRS.

***Material*.** 1♂ (VGPC23807): Mbalmayo Region, Ebogo II village, primary forest 3°23’01.6"N 11°27’50.7"E, 14.VIII.2021, V. Govorov leg.

***Distribution*.** Cameroon, CAR.

***Comments*.** Our samples correspond well with species description, however, after building the phylogenetic tree, the sequence noticeably was placed in the same branch as *Negromantis gracilis* sequence from CAR (MAGAA205). Our suspicion is the specimen from CAR is misidentified.

*Negromantis* sp.

***Material*.** 3♂, 10♀ (VGPC23344-50, VGPC23425, VGPC23491-2, VGPC23808-10): 4♀ Mbalmayo Region, Ebogo II village, primary forest 3°23’01.6"N 11°27’50.7"E, 2-10.VIII.2021, V. Govorov leg.; 5♀, 3♂ same data, but 11-22.VIII.2021; 1♀ same data, but 26.I-9.III.2022, J. Šobotník leg.

***Comments*.** Collected specimens are closely allied with *Negromantis modesta* Giglio-Tos, 1915, but have 8 posteroventral spines on foretibiae. More research is required, including studying of the type material. On the obtained phylogenetic tree, the sequences from this taxon are nesting close together with *Negromantis lutescens* from CAR (MAGA252 and MAGA228), again pointing out possible misidentifications of the latter and urgent need of genus revision.

### Phylogenetic tree and pairwise distances

**Fig 3:**
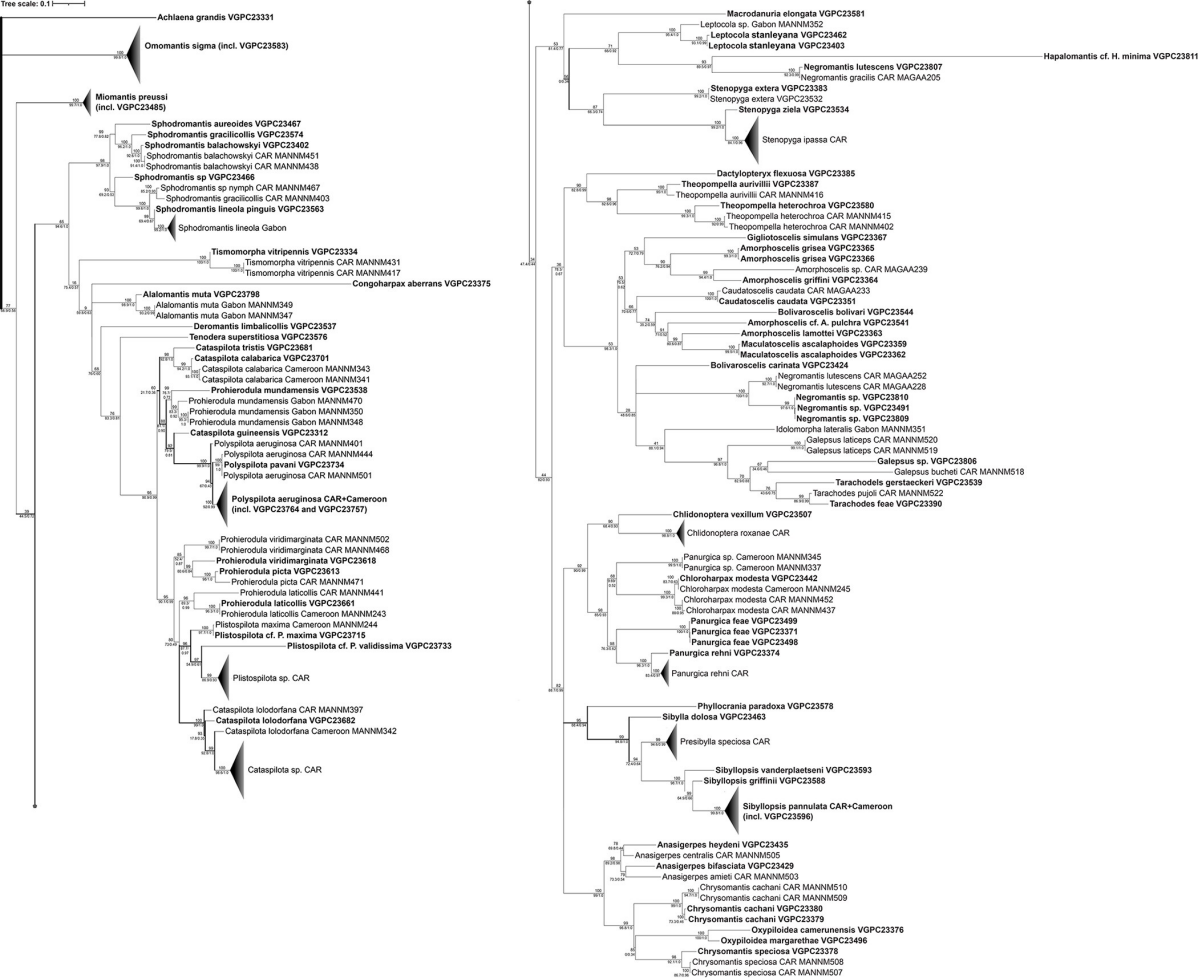
Maximum likelihood tree built using available (n = 165) barcodes of Mantodea of Central African subregion. The number on top of the line is ultrafast bootstrap, two on the bottom are SH-aLRT/aBayes. Barcodes generated during this study are shown in bold.

Maximum likelihood tree was built using sequences from samples collected during our research and joined with available DNA barcodes from CAR, Cameroon and Gabon. The tree (Fig 3) was rooted at *Achlaena grandis*, as according to recent multilocus analysis [41] family Chroicopteridae, to which *A*. *grandis* belongs, is the sister to all taxa in our sampling. Bootstrap supports are given in percent. Maximum likelihood based pairwise distances for all sequences used are presented in S2 Table.

The branches of resulting tree are grouping in accordance with current taxonomy of order [33] in almost all of the cases. Our samples of Dactylopterygidae, Hymenopodidae, Miomantinae, Omomantinae, Tenoderinae, and Tarachodinae are distinctly associated with sequences from CAR and Gabon, while *Congoharpax aberrans* (Galinthiadidae) is nested within Tenoderinae (Mantidae) on its own long branch. Popinae clustered together, however, several samples of Hapalomantinae were also introduced. Same happened in Amorphoscelinae, where *Bolivaroscelis carinata* (Amorphoscelinae) is grouped with *Negromantis* (Hapalomantinae). The situation with split Hapalomantinae likely would be resolved with inclusion of nuclear markers in future phylogenetic analysis.

Barcodes of 6 species (*Bolivaroscelis bolivarii*, *B*. *carinata*, *Polyspilota pavani*, *Sphodromantis aureoides*, *Stenopyga extera*, *Tarachodes gerstaeckeri*) are obtained for the first time. Additionally, two barcodes were obtained for species closely resembling those not currently represented in public databases (*Plistospilota validissima* and *Cataspilota guineensis*), whose precise identification is currently impossible due to the taxonomic state of the respective genera.

## Discussion

The data obtained during this work provide new insights into our knowledge of the diversity of praying mantids in Mbalmayo region and by extension in Cameroon. Previous studies of Roy [20] and Moulin [21] covered collecting efforts in Gabon of several decades, since 1962, resulting in 112 valid species. In contrast, present study is based on two main collecting events in 2021 and 2022 with just few specimens collected at the same site in 2015 and 2018 and yielded 62 species–more than half of 35 years’ worth of inventory of Gabon fauna. Tedrow *et al*. [22] reported results of two surveys in four localities in Rwanda, obtaining 41 species. Moulin *et al*. [23] also report of long-term collecting efforts in CAR, mentioning in their list 71 species, while also providing 94 COI barcodes of African Mantodea for the first time. However, not all species from taxonomic list were selected for DNA extraction, as the authors chose to focus only on southwest area of the country (N. Moulin, pers. comm.), as a result, only 41 species were sequenced. Our study produced 72 sequences and included all species mentioned in checklist, with eight described species receiving a DNA barcode for the first time and five additional are of species likely new to science. Continuous efforts in Cameroon, especially in northern areas in savannah biomes will undoubtably bring country’s checklist on par with neighboring Gabon, as well as help obtain more unique DNA barcodes.

Our research supports the comments of Moulin *et al*. [23] on genus *Plistospilota*, taxonomically complicated genus from the tribe Polispilotini, with original descriptions of the species within this genus are mostly too brief, which makes morphological species identification difficult. At the same time, barcoding seems to clearly separate and group samples into distinct clusters in both Moulin *et* al. and our own analysis. Molecular phylogeny can become a valuable tool in aiding traditional taxonomical methods in revising this taxon, which it desperately needs.

Several collected specimens of Mantodea were not identified to species level. Three males and ten females of the genus *Negromantis* were found to be morphologically closely related to *Negromantis modesta* Giglio-Tos, 1915 but they differed from the original description of the species in number of posteroventral spines on the forefemora. Barcodes obtained from these specimens did not match with any entry in GenBank or BOLD. Considerable differences in barcodes should be evaluated by examining the type material of the genus.

During identification of a specimen of genus *Galepsus*, it was found to have unique genital morphology. While the structure of head vertex fits the diagnosis of the nominative subgenus, none of the species with described copulatory organs possess the same genitalia. Based on external morphology and coloration, this specimen is related to *Galepsus (Galepsus) laticeps* Werner, 1907 but has distinct genitalia. Available sequences of *G*. *(Galepsus) laticeps* and *G*. *(Syngalepsus) bucheti* Moulin, 2018 differ significantly from this sample, with distance being 54.5% and 63.9% respectively. This specimen likely represents a species new to science, however, examination of type material of the species of this subgenus is required.

Several collected specimens of genus *Sphodromantis* resembled quite closely a described taxon, *Sphodromantis lineola pinguis* La Greca, 1967. They have same body shape, spination of the forelegs and very similar structure of genitalia in males. At the same time, these specimens differed in larger body size and most noticeably, pigmented spots on forefemora. Such combination of morphological characters was not matched with any described species. DNA barcode of the sample has the distance of 14% with a sequence of typical *S*. *lineola*, while on phylogenetic tree the unidentified species formed a separate branch with same species. It might be suggested that femoral spots are an early developing character resulted from predator pressure, as the spots are a part of threat display of the mantis. Larger body size might also facilitate in repelling the offenders.

One of the advantages of using DNA barcodes is identification of samples, otherwise impossible to determine due to life stage or sex. This is illustrated on obtained tree in *Stenopyga extera*. One of the samples (VGPC23383) we obtained the sequence from was immature female mantis, belonging to genus *Stenopyga*. However, identification to species-level based on morphological characters was not possible, as the diagnostic characters for the genus include wing coloration (not developed at nymphal stage) and structure of male genitalia (obviously absent in a female) [20]. While building phylogenetic tree, the barcode of immature *Stenopyga* clustered with a sequence from same locality of an adult male *Stenopyga extera*, which was possible to identify based on morphological characters. The distance between two samples is <0.2%, which confirms these two specimens are conspecific.

DNA barcoding proved to be useful in identification of specimens with abnormal morphological traits. One female of *Polyspilota aeruginosa* (VGPC23764) was found to possess blackened prosternum, atypical for this species. Given that *P*. *aeruginosa* is a taxon with high intraspecific variation [24], it is likely an individual aberration. Molecular analysis supports this hypothesis, as the maximum likelihood tree places this specimen close to sequences from CAR, moreover, the pairwise distance between a typical looking sample and the one with blackened prosternum both collected in Ebogo II is about 0.7%. Comparison of the dissected genitalia of this female with a typically looking female revealed no noticeable differences in copulatory organs.

Cameroon is an often-disregarded region when it comes to biodiversity assessments, particularly regarding Mantodea. Our research represents the first comprehensive study on praying mantises of Cameroon, enriching the catalogue of species and enhancing it through the use of DNA barcodes. The use of genetic information allowed us to compare existing phylogeny of the order and to complement existing efforts from neighboring countries. Phylogenetic tree built using sequences of Mantodea of Central African subregion is adequately congruent with current taxonomical consensus. However, several collected specimens likely belong to previously unrecognized taxa, highlighting the importance of expanding the study area and increasing the frequency of sampling to reveal even more undescribed species. The tree, complemented by pairwise distances, demonstrate unresolved taxonomy of several genera, such as *Negromantis*, *Plistospilota*. To address these knowledge gaps, it is crucial to employ a combination of morphological and molecular approaches to fill in the missing data on the biology and ecology of praying mantises in the Central African subregion and, by extension, the entire continent.

## Supporting information

S1 Table Primer pairs used for amplification of the samples and their molecular voucher and accession codes. (DOCX)

S2 Table Pairwise distances. (XLSX)
