# Supplementary material for: First assessment of the biodiversity of praying mantises (Insecta: Mantodea) in Cameroon with DNA barcoding
Source: PLoS One. 2024 May 23;19(5):e0304163. doi: 10.1371/journal.pone.0304163 (PMC11115298; doi:10.1371/journal.pone.0304163)
Supplement: S1 Table — (DOCX) [file pone.0304163.s001.docx]

S1. Table of primer pairs used for amplification of the samples and their molecular voucher and accession codes.

| **Voucher code** | **Binomen** | **Primer pair** | **GenBank accession code** |
| --- | --- | --- | --- |
| VGPC23331 | *Achlaena grandis* | HCO2198/LCO1490 | OR820714 |
| VGPC23798 | *Alalomantis muta* | COH6/COL6b | OR820693 |
| VGPC23541 | *Amorphoscelis cf. A. pulchra* | COH6/COL6b | OR820704 |
| VGPC23364 | *Amorphoscelis griffinii* | COH6/COL6b | OR820700 |
| VGPC23365 | *Amorphoscelis grisea* | COH6/COL6b | OR820684 |
| VGPC23366 | *Amorphoscelis grisea* | COH6/COL6b | OR820683 |
| VGPC23363 | *Amorphoscelis lamottei* | COH6/COL6b | OR820687 |
| VGPC23429 | *Anasigerpes bifasciata* | COH6/COL6b | OR820665 |
| VGPC23435 | *Anasigerpes heydeni* | HCO2198/LCO1490 | OR820723 |
| VGPC23544 | *Bolivaroscelis bolivarii* | COH6/COL6b | OR820703 |
| VGPC23424 | *Bolivaroscelis carinata* | COH6/COL6b | OR820688 |
| VGPC23701 | *Cataspilota calabarica* | HCO2198/LCO1490 | OR820654 |
| VGPC23312 | *Cataspilota cf. C. guineensis* | HCO2198/LCO1490 | OR820713 |
| VGPC23682 | *Cataspilota lolodorfana* | COH6/COL6b | OR820695 |
| VGPC23681 | *Cataspilota tristis* | HCO2198/LCO1490 | OR820722 |
| VGPC23351 | *Caudatoscelis caudata* | COH6/COL6b | OR820680 |
| VGPC23507 | *Chlidonoptera vexillum* | COH6/COL6b | OR820670 |
| VGPC23442 | *Chloroharpax modesta* | COH6/COL6b | OR820671 |
| VGPC23379 | *Chrysomantis cachani* | COH6/COL6b | OR820663 |
| VGPC23380 | *Chrysomantis cachani* | COH6/COL6b | OR820664 |
| VGPC23378 | *Chrysomantis speciosa* | COH6/COL6b | OR820666 |
| VGPC23375 | *Congoharpax aberrans* | COH6/COL6b | OR820711 |
| VGPC23385 | *Dactylopteryx flexuosa* | COH6/COL6b | OR820689 |
| VGPC23537 | *Deromantis limbalicollis* | COH6/COL6b | OR820694 |
| VGPC23806 | *Galepsus sp.* | COH6/COL6b | OR820681 |
| VGPC23367 | *Gigliotoscelis simulans* | HCO2198/LCO1490 | OR820717 |
| VGPC23811 | *Hapalomantis cf. H. minima* | HCO2198/LCO1490 | OR820719 |
| VGPC23462 | *Leptocola stanleyana* | COH6/COL6b | OR820706 |
| VGPC23403 | *Leptocola stanleyana* | COH6/COL6b | OR820708 |
| VGPC23581 | *Macrodanuria elongata* | HCO2198/LCO1490 | OR820660 |
| VGPC23359 | *Maculatoscelis ascalaphoides* | COH6/COL6b | OR820685 |
| VGPC23362 | *Maculatoscelis ascalaphoides* | COH6/COL6b | OR820686 |
| VGPC23485 | *Miomantis preussi* | COH6/COL6b | OR820678 |
| VGPC23807 | *Negromantis lutescens* | HCO2198/LCO1490 | OR820656 |
| VGPC23491 | *Negromantis sp.* | COH6/COL6b | OR820658 |
| VGPC23809 | *Negromantis sp.* | COH6/COL6b | OR820657 |
| VGPC23810 | *Negromantis sp.* | COH6/COL6b | OR820659 |
| VGPC23583 | *Omomantis sigma* | COH6/COL6b | OR820677 |
| VGPC23376 | *Oxypiloidea camerunensis* | HCO2198/LCO1490 | OR820720 |
| VGPC23496 | *Oxypiloidea margarethae* | COH6/COL6b | OR820667 |
| VGPC23371 | *Panurgica feae* | COH6/COL6b | OR820673 |
| VGPC23498 | *Panurgica feae* | HCO2198/LCO1490 | OR820653 |
| VGPC23499 | *Panurgica feae* | HCO2198/LCO1490 | OR820652 |
| VGPC23374 | *Panurgica rehni* | COH6/COL6b | OR820672 |
| VGPC23578 | *Phyllocrania paradoxa* | HCO2198/LCO1490 | OR820718 |
| VGPC23715 | *Plistospilota cf. P. maxima* | COH6/COL6b | OR820696 |
| VGPC23733 | *Plistospilota cf. P. validissima* | COH6/COL6b | OR820707 |
| VGPC23757 | *Polyspilota aeruginosa* | COH6/COL6b | OR820661 |
| VGPC23764 | *Polyspilota aeruginosa* "black prosternum" | COH6/COL6b | OR820662 |
| VGPC23734 | *Polyspilota pavani* | COH6/COL6b | OR820709 |
| VGPC23661 | *Prohierodula laticollis* | COH6/COL6b | OR820698 |
| VGPC23538 | *Prohierodula mundamensis* | COH6/COL6b | OR820702 |
| VGPC23613 | *Prohierodula picta* | COH6/COL6b | OR820697 |
| VGPC23618 | *Prohierodula viridimarginata* | COH6/COL6b | OR820699 |
| VGPC23463 | *Sibylla dolosa* | COH6/COL6b | OR820669 |
| VGPC23588 | *Sibyllopsis griffinii* | COH6/COL6b | OR820668 |
| VGPC23596 | *Sibyllopsis pannulata* | COH6/COL6b | OR820710 |
| VGPC23593 | *Sibyllopsis vanderplaetseni* | HCO2198/LCO1490 | OR820716 |
| VGPC23467 | *Sphodromantis aureoides* | COH6/COL6b | OR820692 |
| VGPC23402 | *Sphodromantis balachowskyi* | COH6/COL6b | OR820691 |
| VGPC23574 | *Sphodromantis gracilicollis* | HCO2198/LCO1490 | OR820715 |
| VGPC23563 | *Sphodromantis lineola* | HCO2198/LCO1490 | OR820655 |
| VGPC23466 | *Sphodromantis sp.* | COH6/COL6b | OR820721 |
| VGPC23532 | *Stenopyga extera* | COH6/COL6b | OR820674 |
| VGPC23383 | *Stenopyga extera* | COH6/COL6b | OR820675 |
| VGPC23534 | *Stenopyga ziela* | COH6/COL6b | OR820676 |
| VGPC23390 | *Tarachodes feae* | COH6/COL6b | OR820682 |
| VGPC23539 | *Tarachodes gerstaeckeri* | COH6/COL6b | OR820705 |
| VGPC23576 | *Tenodera superstitiosa* | COH6/COL6b | OR820712 |
| VGPC23387 | *Theopompella aurivillii* | COH6/COL6b | OR820679 |
| VGPC23580 | *Theopompella heterochroa* | COH6/COL6b | OR820701 |
| VGPC23334 | *Tismomorpha vitripennis* | COH6/COL6b | OR820690 |
